# On Convulsions in Women during Pregnancy and Labour, and after Delivery

**Published:** 1835-07-01

**Authors:** 


*Des Convulsions chez les Femmes, &c.*

*On Convulsions in Women during Pregnancy and Labour, and after Delivery.* By A. Velpeau, Surgeon to the Hôpital de la Pitié. Paris and London, 1834. 8vo. pp. 132.

M. Velpeau has long been distinguished for the determined and successful industry with which he has laboured at various branches of medical science; but he had yet to afford a proof of the remarkable facility he possesses, in giving to the profession the result of his labours. At a recent *Concours* in Paris, he was required, at a very short notice, to prepare a thesis on the subject of Puerperal Convulsions, and the really wonderful rapidity with which he furnished an excellent essay, abounding in well-arranged practical facts, and numerous illustrations from the best authorities, proved that his promptitude is equal to his perseverance. The work before us is an amplification of the thesis; and we shall at once proceed to impart to our readers the sum and substance of the author's opinions.

In the first chapter are discussed the different forms and frequency of puerperal convulsions.

Experience teaches us, that women may be attacked during pregnancy, in the act of labour, and after delivery, with the same kind of convulsive disease. The apoplectic form is more uncommon, however, during pregnancy than during labour: the hysterical variety more frequent. Partial convulsions more generally occur before and after delivery, than at the time of childbirth. As in the unimpregnated female, the convulsions which occur during pregnancy may either be general or local. Either one or all the limbs may be affected; the face, or some other part of the body, may suffer alone, or all the locomotive muscles may be simultaneously convulsed. The voluntary muscles are most subject to convulsions; but, in some cases, the viscera are not exempt. Levret,* for example, mentions a species of convulsions, which attacks only the solid muscles and the sphincters of hollow muscles. Thus, as is known to every medical observer, the pharynx, the œsophagus, the stomach, the intestines, the uterus itself, the heart, and particularly the diaphragm, are sometimes attacked with violent convulsive action. Partial convulsions, however, are by no means common; and we agree with M. Velpeau in the belief that many of the examples that are recorded might fairly claim some other denomination. As such a form of the disease is rare, well-authenticated instances of it are propor-

* Art des Accouchemens, &c. troisième Ed. p. 233.

tionately interesting. We, therefore, give the following case, which was communicated to M. Velpeau by M. P. Dubois.

A woman, about the fifth month of pregnancy, was attacked with a very singular convulsive affection of the abdomen. The parietes of the belly contracted with so much violence, that the uterus was pressed into the pelvis. Immediately afterwards, this organ suddenly resumed its former situation, rebounding like an elastic ball thrown upon the ground. Irregular contractions also took place in the groins, the epigastrium, and the umbilical region, which appeared to depend upon spasmodic contraction of the viscera as much as of the ventral parietes. The patient recovered, and she did not miscarry.

Denman, we may state, mentions a somewhat similar fact. He says,* that, in a few cases, he has known the action of the abdominal muscles so regular and strong, that the whole volume of the uterus has been heaved up and down, alternately, in such a manner, that it was scarcely possible to distinguish between this strange succession and the proper action of the uterus; yet without any dilatation of the os uteri. The viscera alone are more frequently affected with these morbid contractions. The following is a remarkable case.

A female, aged twenty-two, of a rigid and nervous constitution, was delivered naturally, of her third child. She attempted to get up and work on the seventh day after labour; but she was compelled to return to bed, on account of severe pains in her belly. On the tenth day, she was alarmed at certain movements which she felt in the abdomen, and which increased every moment in violence. M. Velpeau was now consulted, and was as much astonished at what he witnessed as the patient herself. He saw, through the abdominal parietes, a sort of moveable ball, which traversed every part of the belly; sometimes it moved towards the pelvis; then to the groins; then to the navel. This ball was sometimes changed into several projections, which rolled about the abdominal cavity with a noise; the parietes of the belly always preserving their normal laxity. The patient was quickly impressed with the belief that she had an animal in her body, and that she was "*vouée à l'enfer.*" Her mind gave way, and she became mad. She was admitted into the hospital at Tours, and died two years afterwards; the singular movements above described never having entirely ceased. Upon dissection, the peritoneum and muscles were found as black as ebony, "*quoique sains*"*!* in the hypogastric region. The digestive organs healthy. The uterus was thickly studded with fibrous tumours. One of the ovaries had degenerated into a multilocular cyst, and the corresponding Fallopian tube presented a similar appearance. The brain, lungs, and heart, were healthy.

* Denman's Midwifery; edited by Dr. Waller; p. 192.

Smellie and Plenck* assure us they have found the vagina so contracted by convulsion as to prevent the exit of the child. Halma Grand† and Mondiere‡ say that they have found the vagina so contracted as to benumb the hand of the practitioner during the labour. "*But* it is not clear that some mistake has not been committed in these cases."

*Partial* convulsions of the uterus are most interesting to the accoucheur, and examples of this form of disease have been frequently witnessed, during pregnancy, at the time of labour, and after delivery. M. A. Menard§ mentions an instance, in which the uterus assumed the form of a gourd. M. C. Baudelocque|| and Deneux¶ relate another, in which the womb rose and fell, and was carried first to the right and then to the left, with surprising force. M. Ed. Petit** affirms, that, in the case he saw, the convulsions of the uterus were so violent, that at each moment it was precipitated towards the vulva, and that he was obliged to support it with his fingers, to prevent its expulsion. If we admit that a little exaggeration may enter into these details, still the facts have been recorded by contemporaries of too much character to justify our disbelief in their statements.

Convulsions of the uterus during the act of labour are more easy to comprehend, as, at the time of the expulsion of the child, each uterine contraction partakes of a convulsive character; the contractions sometimes pervade the whole of the organ, but generally only one portion of it, especially the cervix, either at the vaginal or uterine orifice. After the delivery of the child, too, we often have spasmodic action of the uterus, impeding the exclusion of the placenta.

*Varieties of Puerperal Convulsions.* As puerperal convulsions present different phenomena in different cases, which resemble other convulsive affections that attack men, and women who are not pregnant, various terms have been applied by writers to mark these distinctive forms of the disease. Thus, mention is made of the tetanic, cataleptic, hysterical, epileptic, apoplectic, and choreal. Dr. Merriman†† speaks of puerperal convulsions as always assuming the appearance of epilepsy; and this we believe to be the most common, but

* Art des Accouchemens, p. 122. Miguel, p. 156.
† Gaz. Med. de Paris, 1831, p. 323.
‡ Des Rupt. de l'Uterus. Mem. de la Soc. d'Emulat. 1834.
§ Transact. Med. t. iv. p. 246.
|| Thése sur les Convulsions; &c. n. 84. Paris, 1822.
¶ Miguel; p. 108.
** Bibliot. Med. t. xxix. p. 171. Baudel. Th. p. 82.
†† Synopsis of difficult Parturition; p. 139. 4th Edit.

certainly not the only form, of the disease. Vogel, too, regards it as an acute epilepsy. Burns* asserts, however, that "puerperal convulsions are quite different from epilepsy;" and that the most frequent species is of the nature of "eclampsia, or of tetanus, which occurs a hundred times, for once that the others appear." This by no means accords with our own observation. Dewees,† who wrote especially on the disease, in 1818, retains three forms, the epileptic, hysterical, and apoplectic.

M. Velpeau agrees with Mdme. La Chapelle,‡ that the convulsions of pregnant women differ in most cases from either of the above-named diseases, from which other writers have drawn the titles of the varieties of puerperal convulsions; and he thinks, with Desormeaux,§ that it is better to use the term *Eclampsia,* unless that of *Dystocia Convulsiva,* employed by Dr. Young, should be preferred. Menard‖ states that the *aura epileptica* generally precedes eclampsia; but M. Velpeau denies the accuracy of this opinion: and he further states, that it is proved by a crowd of facts, (and one, the case of a lady, fell under his own observation,) that pregnancy repels or suspends the paroxysms of simple epilepsy. The great use of a term is to convey so definite a meaning that no one can misapprehend it: now we fear that many readers will not know at all what *eclampsia* means; while others will be aware that it is one of those numerous terms, whose signification has been so altered at various times, by different nosologists, as to leave us in great doubt as to what is really implied by it.¶ In every point of view, therefore, we should prefer the *Dystocia Convulsiva* of Young.

We gain no very satisfactory information as to the frequency of puerperal convulsions, in a given number of pregnancies, by consulting the different statements that have been published; so greatly do they vary. It appears highly probable, however, that in some years, and in certain states of the atmosphere, the disease occurs more frequently than others. La Chapelle tells us, and other writers confirm the fact, that, at the Maternité, it sometimes appeared as an epidemic, and that one woman was rarely attacked without other cases speedily following. The period of its greatest frequency is incontestably the time of labour; not at the beginning of the parturient

* Midwifery; p. 483. 8th Edit.

† Essay on Puerperal Convulsions.

‡ Pratique des Accouch. t. iii. p. 16.

§ Dict. de Med. t. vii. p. 292.

‖ Transact. Med. t. iv. p. 245.

¶ Vide Dict. des Sc. Med. t. ii. Art. *Eclampsia.*—REV.

process, nor at the end of it, but during the long period that divides the two. Puerperal convulsions, in the strict acceptation of the term, rarely occur before the sixth month of pregnancy, and it is correctly stated by Burns* that the convulsive attacks which occur during the four first months of utero-gestation, assume the character of hysteria, and cause but little anxiety.† Exceptions occasionally arise, however, to this very general rule. A young woman, for example, was attacked with convulsions in the fourth month of pregnancy and the case ended fatally. Willis mentions a similar instance.‡ After delivery, too, this disease is still less frequent. In thirty cases that occurred at the Maternité, not one took place after the birth of the child.

After these preliminary observations, M. Velpeau gives a brief practical detail of twenty-four cases: some of them occurring during pregnancy, and others during and after labour.

The causes of Eclampsia—we retain the term employed by M. Velpeau—are very numerous, and may be divided into the predisposing and occasional. Women of a strong plethoric habit and rigid fibre, with an animated countenance, and short neck, and those also who menstruated profusely, and are of a nervous, delicate, and irritable frame, of youthful age, and in first pregnancies, are more exposed to the disease than others. The cases related by M. Velpeau prove, however, the accuracy of the general opinion that the disease not unfrequently occurs in the second, third, fourth, and even sixth pregnancies. In one case, mentioned by Dumont,§ the patient was attacked in her eleventh pregnancy. Particular states of the stomach and bowels appear also to favour the development of convulsions; and hence, no doubt, the partiality of many practitioners for emetics and purgatives in their treatment. Velpeau himself is inclined to confine this cause within very narrow limits. Œdema of the lower extremities is another cause of eclampsia, that has attracted the notice of practical men; and M. Velpeau and Mdme. La Chapelle|| confirm the general opinion.

*During pregnancy*, the great modifications which conception causes in the whole nervous system of many females evidently

* Loc. cit. p. 238.

† Our practical readers will agree with us that the following declaration of Jörg's is very remarkable from so experienced and close an observer. "Nie habe ich aber die Hysterie an Schwangern und Säugenden wahrgenommen. Krankheiten des Weibes; p. 255. Leipzig, 1821.

‡ Encyclop. Méth. t. ii., p. 248.

§ Journal Général; t. iii. p. 482.

|| Loc. cit. t. iii. p. 8.

predisposes them to convulsive attacks during the early months of utero-gestation: but, then, the convulsions are purely nervous, and rarely present the characters of true eclampsia. The suppression of the menses, which necessarily retains in the blood certain materials that were destined to be expelled, increases the irritable disposition of pregnant women, in the very early months of pregnancy, when the product of conception requires but little vascular support from the mother. The uterus, gorged with blood, becomes the seat of important actions, and reacts powerfully upon the whole economy through the nervous system. The vomitings and palpitations, which the slightest causes produce, prove how easily convulsions may be produced. The volume which the uterus acquires at a later period of pregnancy, gives rise to still more striking derangements. The pressure inflicted upon the blood-vessels and nerves of the pelvis, and even upon the aorta, produces such changes in the circulation and transmission of the nervous influence, that we cannot be surprised at local congestions and cerebral excitement.

*During labour,* eclampsia is rendered likely, in M. Velpeau's opinion, by extreme distention of the uterus, and by the obstruction which the fluids must experience in passing through the organ; by rigidity, and spasmodic contraction of the cervix, excessive sensibility of the whole of the womb; or by a bad position of the child, or any mechanical impediment, which may retard delivery. The general irritation communicated to the whole system during the expulsive period of labour easily passes into general or partial convulsions.

Immediately *after labour,* the woman remains under the influence of as important modifications as during the act of labour. The quick depletion of the abdomen suddenly changes the locality of all its viscera. The blood, which before traversed with so much difficulty the inferior parts of the aortic system, now rushes through in full streams, and with the more freedom as the viscera are now unsupported. Pregnancy and labour excite the encephalo-spinal system by determining to it an increased quantity of the vital fluids. Delivery disturbs the functions of this important apparatus, by depriving it too suddenly of its natural stimulus; and, after the general disturbance produced by gestation and labour, the natural tendency to restore the equilibrium is not excited without giving a new shock to the nervous power.

The *occasional causes* of eclampsia we shall not formally notice. Many that enter into the list of systematic writers are more imaginary than real; in fact, whatever circumstance happens to precede the attack is noted as the occasional cause of the disease, although it is quite certain that, in many cases, no satisfactory reason can be assigned for its occurrence.

*Symptoms and Progress of Eclampsia.* In *some* women, says M. Velpeau, (*we* should *at least* have said, in *most*,) the paroxysm is announced by various premonitory signs, as great heat of the head, giddiness, confused mind, impeded motion, and uneasiness of the limbs; a vacant, or frightened look; turgid conjunctiva, or turgescence of the whole face; swelling of the neck and face;* headach; imperfect speech; irregularity of pulse; convulsive movements of the muscles; subsultus tendinum. A woman, who was admitted into the Maternité with these premonitory symptoms, was thought to be intoxicated by the students. Very often, however, the disease makes its attack suddenly and unexpectedly, and at once is marked by the most alarming symptoms. Deleurye† and Dewees‡ are wrong, in M. Velpeau's opinion, in their belief of the *constant* occurrence of the preliminary symptoms. We do not contend that the paroxysm *is always* to be thus anticipated; but our own experience teaches us the truth of the general doctrine, that it very rarely happens, if we have the opportunity of fair and full inquiry, that some symptoms may not be detected which usher in the fit of convulsions. The pain in the head, upon which Dewees particularly insists, and which Puzos and Hamilton had before noticed, M. Velpeau admits as a frequent premonitory symptom. Dewees remarks,§ "that, the longer the premonition, the milder the attack appears to be; and on the contrary. The most suddenly fatal case we ever remember to have seen, was where the patient suddenly cried out "my head! my head!" convulsions instantly ensued, of which she died in a few hours."

Denman, and other writers, attach much importance to pains in the stomach; and, in his opinion, when convulsions are thus announced, they are more severe than when they are preceded by pain in the head. Both these symptoms have been observed by M. Velpeau; but in most of his cases they were wanting. It is singular that Chaussier and Mdme. La Chapelle, who drew their inferences from the practice at the same establishment, have given contrary opinions as to the

* J. F. Osiander states that he has seldom observed a tumid state of the face and hands wanting as a premonitory symptom.—Rev.

† Art. des Accouch. &c. p. 136.

‡ Midwifery, p. 498. "Convulsions (puerperal) are all preceded by symptoms which denote their approach." Chaussier thinks they are scarcely ever wanting altogether.—Rev.

§ Midwifery, 498.—Rev.

frequency of the precursory signs of puerperal convulsions. We have already stated that the former asserts they are almost always present: the latter* says they are frequently wanting. Some women, says M. Velpeau, feel a weight in the hypogastrium, or hardness, or even a painful sensation, even weeks, or days, or some hours, before the attack. In three instances, he has known this symptom exist in a strongly-marked manner, and he thinks it has been too much neglected. If the hand is laid upon the uterus, through the parietes of the abdomen, it is felt in a state of tonic contraction, firm and sensible upon the least pressure. The symptoms of a violent paroxysm are most graphically described by the author. The duration of the fits is as various as its intensity. It may pass off in a few minutes; it may last an hour or more; and even twelve or twenty-four hours may elapse before the patient regains her consciousness; and, in some cases, if coma does not take place, the loss of consciousness may be prolonged for several days, and still the patient promptly and perfectly recover. The paroxysm is generally repeated; and the succeeding attack is often indicated, according to Croft and Merriman,† by decided slowness of the pulse. M. Velpeau has noticed this curious phenomenon. Sometimes the paroxysm assumes all the characters of apoplexy, and death ensues. In a case we saw for Dr. R. Lee, the patient, a very fine young woman, of a full plethoric habit of body, presented during the fit so completely the ordinary appearances of profound apoplexy, that no practitioner could have distinguished between the two diseases. She died in a few hours, although the treatment adopted was, we believe, judicious; it was certainly promptly applied. In some instances, after the cessation of the paroxysm, certain functions remain perverted. The sight, hearing, or smell, or some of the intellectual faculties, may be injured. Internal ruptures, local extravasations of blood, have also been caused. Dislocations of the jaw have taken place.

After having described the more ordinary and epileptic form of the disease, M. Velpeau briefly notices the distinctive symptoms of the more uncommon varieties; as the hysterical, the tetanic, and apoplectic.

*Termination and Prognosis.* Puerperal convulsions may terminate either in health, by the death of the patient, or by the production of some other disease. We may expect a favourable result when the paroxysms become more and more distant, or by their becoming shorter, even if their frequency

* Loc. cit. t. iii. p. 8.
† Loc. cit. 4th Edit. p. 140.

continues. The torpor and coma gradually subside, and the woman appears to rally from a long disease. On the contrary, if the paroxysms increase in length and severity; if the comatose symptoms are more decided than the convulsive phenomena, death is to be feared; and the fatal event may take place after different durations of the disease, varying from half an hour to many hours. It appears, from abundant testimony, that eclampsia has frequently produced rupture of the uterus. Deneux* appears even to admit that most ruptures of the uterus depend upon partial convulsions of the organ. The nature of the symptoms evinces the milder disease that sometimes results from the convulsive attacks. Hemorrhage within the brain is the most to be feared; and hence, no doubt, the reason why the disease has by several writers been confounded with apoplexy. We do not believe that any case of puerperal convulsions, if seen from the commencement, could be mistaken for apoplexy, but, as we have before had occasion to remark, all the symptoms of the latter malady sometimes arise in the progress of the former; and then, of course, the distinction between them is lost. The sudden determination of blood to different viscera, and the violent and unequal pressure to which different organs are exposed during the convulsive struggles, expose the patient also to many congestions and inflammations. Mdme. La Chapelle† states that many women, affected with eclampsia, die afterwards of peritonitis; and M. Cinisellli‡ has recently published a case which confirms the opinion of this distinguished authoress.

The *Prognosis* is generally unfavourable both for the mother and child. Mdme. La Chapelle§ confesses that, in spite of the best and most decided treatment, about half the cases at the Maternité terminated fatally. Hunter‖ and Lowder express the same opinion.¶ Dr. Merriman, whose personal experience upon this point is *not* given by M. Velpeau, although most of the authorities mentioned by him *are* quoted, states that, "in modern practice, the proportion of deaths is by no means so great." In forty-eight cases, attended by Dr. M. in private practice or in consultation, thirty-seven women recovered, and eleven died; and be it observed, too, that Dr. M.

* Miguel, op. cit. p. 108.
† Loc. cit. t. iii. p. 21.
‡ Annal. Univers. de Med. t. 49, 471.
§ Loc. cit. t. iii. p. 18.
‖ Loc. cit. p. 139.
¶ Burns says (Midwifery, 8th Edit. p. 489,) "If the practice be prompt and vigorous, the generality of patients recover from puerperal convulsions!" We wish the learned professor had given a circumstantial account of the evidence upon which this opinion is founded.—REV.

says, in some of these cases "the plan of treatment was not always so early and effectually adopted as I could have wished." The result of these cases is highly creditable to Dr. Merriman. The disease is less fearful, M. Velpeau states, *cæteris paribus*, during than before labour; and, as delivery is frequently the only means of relieving the symptoms, it is evident that the danger of the convulsions must therefore be in direct proportion to the difficulty of effecting delivery. During labour, eclampsia sometimes terminates by the sudden expulsion of the child. When the disease attacks nervous and hysterical females, it is less hazardous than when it occurs in plethoric subjects. The more apoplectic the character of the attack, or its progress, the greater the danger. The danger to the child is still greater than that of the mother: it frequently dies in the midst of the convulsive struggles. In artificial labours, even at the full period, the child generally dies when the mother is convulsed; but, if the delivery can be promptly effected, the child may be saved.

*Appearances after death.* Our *post-mortem* examinations are far from always accounting satisfactorily for the severity of the symptoms during life. The substance of various statements from the best authorities upon this subject confirms almost entirely the opinion of Mdme. La Chapelle, "that, if apoplexy is not combined with the attack, the organic alterations are not in relation to the intensity of the symptoms."* It is to be lamented that the spinal marrow has not been more frequently examined, that we might be enabled to ascertain the accuracy of the opinions expressed by Burns,† and others, as to the pathology of the disease.

*Treatment.* No one mode of treatment can be expected to succeed in so variable a malady. With respect to *antispasmodics*, M. Velpeau states that, if they are not proved to be serviceable in the epileptic variety of eclampsia, they certainly are in the hysterical; but they rarely are sufficient alone, even in this mild form of the disease. They moderate rather than dispel the paroxysm, and their efficacy must not be too much relied on, even in partial convulsions. Large doses of camphor, to which some practitioners are so partial, M. Velpeau would rarely administer except in clysters.‡

*Narcotics.* By most practitioners, the use of opium is altogether condemned in this disease. Bland,§ however,

* Loc. cit. t. iij. p. 25.
† Midwifery, p. 48.
‡ Dr. Copland and others speak very favourably of the effects of camphor. See Copland's Med. Dict. art. Convulsions, 433.
§ Observations on Parturition; p. 138.

regards it as an heroic remedy, to which no other can be preferred. We believe, upon this very important practical point, that M. Velpeau is quite correct in thinking that both parties have carried their opinion too far. "Reason and analogy, rather than experience, at first indicated the employment of opium. Its reputation of favouring cerebral congestions induced its opponents to reject it from the treatment of a disease which is frequently characterized by that condition of the brain. But, in fact, it merits neither the abuse nor the eulogies that have been lavished upon it; it is an auxiliary that ought not to be neglected, when there is neither stertor nor comatose symptoms." We apprehend that no rational practitioner would ever attempt to relieve a convulsive disease by opium, if it were attended by either stertor or coma, however slight might be the degree. Hysterical eclampsia, and all convulsions originating in spasm, distention or irritation of the uterus, have appeared to M. Velpeau to be relieved by opium, after venesection had been practised, or when it was not indicated. In common with the prevailing opinion, he looks upon this remedy as useless, and even dangerous, in the apoplectic, or even epileptic forms of the disease. He greatly prefers the preparations of morphia, especially the powder, in doses of a quarter or half a grain every two or three hours, in a glass of cold water, when this class of remedies is deemed advisable. "They are less likely to produce congestions, and are quite as efficacious as sedatives, as the extract or tincture of opium." He thinks well of the local application of opium, in the form of pomade, to the cervix uteri, during labour, in partial convulsions of the womb. In *general* convulsive attacks this mode of applying the remedies would be of little avail. Laudanum has been also injected into the uterus, but we want facts in support of the practice; and the same objections apply to it as to the internal exhibition of opium. Belladonna has also been employed in puerperal convulsions both internally and locally to the os uteri; and it appears, from the evidence of Evers, Chaussier, Conquest, &c., with occasional advantage.

*Evacuants and Emetics* have been much eulogised. "They can rarely be given during labour, unless the convulsions appear to depend upon an overloaded stomach; but I would willingly have recourse to them during pregnancy and after labour, if the symptoms appeared to have the least connexion with derangement of the digestive organs, especially the stomach, whether the attack partook of the epileptic or apoplectic form. I should be more averse to the use of these remedies in hysterical convulsions." (P. 34.)

*Purgatives.* Of these Dr. Merriman thinks very highly:* M. Velpeau considers them less efficacious because their operation is slower than emetics. The former may, however, be given with less hazard than the latter. Stimulating injections "can neither be very prejudicial nor very useful." To this opinion we find Dr. Copland is opposed.†

*Bleeding* is the remedy principally relied upon by most practitioners: it is the only one in which Mdme. La Chapelle has much confidence.‡ Gooch,§ too, we may add, is thus reported to express himself in his Lectures: "Give me the lancet, and deprive me of all other remedies, and I will do more good with it singly, than with all others, deprived of this, put together." The abstraction of blood has been frequently and effectually employed to *prevent* an attack of puerperal convulsions, when the premonitory symptoms warned the practitioner of the impending danger. Still we think—nay we are sure, that M. Velpeau is quite justified in hesitating to admit the assertion of Dr. Davis, Deleurye, &c., who declare that bleeding to the amount of forty or fifty ounces will always prevent the attack; and that, if convulsions occur during labour, it is a slur upon the skill of the practitioner. How difficult the proof must be. A woman, apparently threatened during her pregnancy with convulsions, is freely bled. No convulsions take place; but it would be very bad logic to declare positively that they would have taken place, if the lancet had not been used. We are very far from denying the frequent propriety of bleeding as a preventive measure, but we cannot admit the infallible success of the remedy; for convulsions in the puerperal states may occur from *great exhaustion,* from want and inanition, and *losses of blood;* and, in such instances, too, the convulsive struggles are as severe, if not more so, than when a plethoric and robust patient is attacked. Chaussier|| himself, one of the warmest advocates for bleeding, either as a remedial or preventive measure, in puerperal convulsions, gives the following case. A young female complained of severe pain in the head in the ninth month of her first pregnancy: she was largely bled. Two days afterwards, she fell into violent convulsions, and died in twenty-one hours. As a curative measure bleeding has been had recourse to in every form, and experience tells us that sometimes the remedy fails,

* Loc. cit. p. 143.

† Loc. cit. p. 435.

‡ Loc. cit. t. iii. p. 29.

§ Compendium of Midwifery, by Skinner ; p. 245.

|| Des Convulsions des femmes enceintes, p. 8.

sometimes it succeeds, or at least the patient recovers. In two instances, we have ourselves seen it fail to make the slightest impression upon the disease; in both the patients died. Bleeding from the veins of the feet has had many partisans on the continent; but this mode of depletion has now nearly fallen into disuse. Blood should be drawn, in preference, from the jugular vein, or from the arm. Cupping may be employed as an auxiliary, but rarely as a principal agent. Many practitioners recommend very copious bleedings. The quantity of blood that some do not hesitate to abstract appears, indeed, alarming. Dewees, for example, speaks of a patient who lost ninety-seven ounces in seven bleedings; and of another, who recovered, and who lost 120 ounces in the first six hours, and 140 ounces altogether.* M. Velpeau states some facts which prove the danger of these enormous bleedings. Much must, of course, be left to the judgment of the practitioner; but we cannot forbear from remarking, that a patient may be bled *into* convulsions or apoplexy, as well as *out* of them. He would, with Cruveilheir, proscribe so free a use of the lancet, and recommends, in preference, smaller and repeated bleeding, according to the indications. And, lastly, upon this point M. Velpeau questions whether general bleeding justifies its frequent employment. From the appearance of the symptoms, we should answer in the affirmative; but our confidence must be shaken to a certain extent, when we find that Mauriceau and Mdme. La Chapelle, who bled largely, lost half their patients; whilst Dr. Merriman, who bleeds much less, has rescued a much greater proportion.

It is not to be supposed that rather mild cases of eclampsia would always terminate fatally without bleeding. Case 10 and 15, related by M. V., are proofs to the contrary; but in these instances, we must add, the disease appears to have assumed more of the hysterical than of the severer forms of the disease.

Revulsive remedies, as sinapisms, blisters, dry-cupping, &c., have also occasionally a place in the treatment. Cold aspersions upon the face, of which Dr. Denman speaks so highly, are, in the opinion of the author, of use only in the hysterical form of eclampsia. But iced water, applied to the forehead and head, he thinks highly of; and his opinion is confirmed by many of his most distinguished countrymen. Here, too, we look upon cold applications to the head as ne-

* M. Velpeau, by mistake, misquotes Dr. Dewees. Dr. D. states that the patient lost 140 ounces of blood *altogether*; not "cent vingt onces dans les six premières heurs, et cent quarante onces ensuite." This would be 260 ounces altogether. See Dewees's Midwifery, p. 506.—Rev.

cessary and useful, especially where there is much pain in the head and cerebral irritation.

Besides these general remedies, eclampsia requires also other resources, according as it occurs during pregnancy, labour, or after delivery: but, for M. Velpeau's opinions upon the modified treatment required in these different conditions, we must refer to the work, the perusal of which will amply repay any student or practitioner who wishes to become acquainted with the opinions entertained by the best authorities respecting the pathology and treatment of one of the most interesting, formidable, and (may we not still add?) obscure diseases incidental to puerperal women. The condensed yet lucid style, and impartial manner, in which M. Velpeau has collected information from a great variety of sources, would alone render his monograph very valuable. It is additionally so, because he has freely incorporated his critical remarks and the results of his own experience.

